# Editorial: The Circadian Clock and immune system interplay in infectious diseases

**DOI:** 10.3389/fcimb.2022.1036633

**Published:** 2022-10-25

**Authors:** Mariela Chertoff, Luciano Marpegan, Nicolás Pregi, Natalia Paladino

**Affiliations:** ^1^ Laboratorio de NeuroEpigenética, Departamento de Química Biológica, Instituto de Química Biológica de la Facultad de Ciencias Exactas y Naturales (IQuiBiCEN), Facultad de Ciencias Exactas y Naturales, Universidad de Buenos Aires, Consejo Nacional de Investigaciones Científicas y Tecnológicas (CONICET), Buenos Aires, Argentina; ^2^ Departamento De Física Médica, Comisión Nacional De Energía Atómica (CNEA), Consejo Nacional de Investigaciones Científicas y Tecnológicas (CONICET), Buenos Aires, Argentina; ^3^ Laboratorio de Cronobiología, Departamento de Ciencia y Tecnología, Universidad Nacional de Quilmes, Buenos Aires, Argentina; ^4^ Consejo Nacional de Investigaciones Científicas y Tecnológicas (CONICET), Buenos Aires, Argentina

**Keywords:** circadian rhythms, infection, immune system, microorganism, circadian disruption, bacterial infection, viral infection

Animals must interact with their environment exposing themselves to harmful chemical, physical, and biological threats. Far from being constant, the milieu in which most animals thrive is extremely variable with most of such variations occurring as daily cycles. So they must obtain what they need while defending themselves from external threats within strict temporal niches. The current Frontiers Research Topic brings together recent breakthroughs on how the defense mechanisms (immune system) and the entraining mechanisms (circadian system) interact to defend the organism from infectious diseases.

The circadian system is responsible for the generation and entrainment of rhythms for almost all physiological and behavioral functions of the body (e.g., body temperature, hormonal secretion, sleep, and locomotor activity), enabling adaptation to cyclic environmental changes. A cell-autonomous pacemaker mechanism generating close to 24h cycles based on negative transcriptional-translational loops was found in almost every cell type within the body. A small group of neurons residing in the hypothalamic suprachiasmatic nuclei (SCN) sets the pace for the rest of the body while receiving input from the retina to entrain themselves to the external light-dark (LD) cycles. The SCN controls circadian oscillators in peripheral organs through neural, hormonal, and behavioral pathways to maintain an optimal phase relation between them and with the environment. In addition, the endogenous clock in peripheral tissues is entrained by behavioral cues such as sleep-wake cycles, food intake, and physical activity, independently of the SCN. In humans, shifting the activity period to an atypical time of the day (e.g., by shift-work or jet-lag) causes a temporal misalignment that may lead to the onset and progression of multiple diseases, including obesity, type-2 diabetes, cardiovascular diseases, cancer, systemic inflammatory diseases and psychiatric disorders (Golombek et.al., 2013).

Circadian modulation of the immune system has been studied under basal conditions and under pathogenic challenges. Daily fluctuation of the concentration of circulating leukocytes, hormones, cytokines, and chemokines regulate innate and adaptive immunity (Cermakian et. al., 2013) allowing organisms to anticipate daily variations of behavioral activities and thereby risks of infection and tissue damage.

Taleb et al. and Mul Fedele et al. describe how the immune response, severity and outcome of infectious disease in murine models show temporal dependency. Taleb et al. (2022) showed, in a murine model of Inflammatory Bowel Disease (IBD), time differences in disease severity, epithelial proliferation, immune cell infiltration and TNF-α levels in the colon. Similarly, Mul Fedele et al. describe the differential outcome observed in sepsis murine models, and the daily pattern of macrophages and TNF-α levels. They both show circadian disruptions not only abolished the observed daily patterns, but also worsened prognosis and impaired recovery. In addition, Shirato and Sato reviewed temporal modulation in several respiratory tract infections, including SARS-CoV-2, and described the circadian regulation of activated immune pathways.

Chronic circadian misalignment causes dysregulations of the innate immune system leading to altered responses such as an increased release of proinflammatory cytokines during endotoxic shock (Mul Fedele et al). As explained by Shirato and Sato and Mul Fedele et al., macrophages play a central role in circadian regulation of the immune response. These cells gate the timing not only for the defense against bacterial and viral infections, but also for tissue repair and regeneration. Macrophages are critical players in the inflammatory response which if uncontrolled might cause serious tissue damage. In this regard, Shitaro and Sato provide detailed information on how inflammatory response related gene expression (i.e. cytokines and chemokines), is modified due to misaligned circadian systems induced by jet-lag, shift work or the time of infection. At the molecular level, mouse models for circadian disruption show a dysregulation of inflammatory gene expression in macrophages. Specifically, macrophages harvested from animals under circadian disruption exhibit a robust response to LPS stimulation. These data point directly to the essential contribution of macrophages to the development of systemic inflammatory status following chronic circadian misalignment.

In addition, macrophage functions can be fine-tuned through rewiring of metabolic pathways. Interestingly, the feeding time modulates sepsis prognosis and mitochondrial metabolism also regulates the activity of macrophages as described by Shirato and Sato.

Regarding anti-inflammatory mechanisms, glucocorticoids are particularly relevant. Their serum levels peak at the beginning of the active phase and drop during the resting phase. The robust circadian oscillation of circulatory glucocorticoids modulates inflammatory cytokine and chemokine expression leading to cyclic activity of systemic inflammation and immune response. As mentioned by Mul Fedele et al., the role of glucocorticoids in sepsis treatment is controversial. A possible reason for this is the lack of circadian considerations in most of the reports.

The interaction between the circadian and immune systems is reciprocal. As mentioned above, the circadian system modulates various aspects of the immune system while inflammation mediators can modulate the circadian rhythms and sleep. Zielinski and Gibbons exhaustively described the effects of different cytokines on sleep and slow-wave activity (SWA). They explain how proinflammatory cytokines (IL1β and TNF-α) promote sleep and SWA while antiinflammatory cytokines (IL-4, IL-10, IL-13, and IL-1RA) attenuate sleep and SWA responses induced by sleep deprivation or pathogenic components. Inflammatory molecules that regulate sleep can affect clock genes and vice versa, which likely contributes to an overall enhancement or suppression of sleep pressure. In this regard, Taleb et al. showed that IBD dampens circadian rhythms in the colon possibly by suppressing Bmal1. However, under certain conditions, it could induce a vicious cycle in which circadian disruption hinders the development of an adequate immune response.

Taken together, this Research Topic covers the main aspects of the interplay between the immune system and circadian clock in infectious diseases, showing that the outcome of a disease is not only related to how pathogens interact with the host but also to when this interaction happens and the state of the circadian system. In a world that has lost track of natural time, the relevance of circadian disruption on disease progression and treatment is fundamental.

## Author contributions

All authors listed have made a substantial, direct, and intellectual contribution to the work and approved it for publication.

## Conflict of interest

The authors declare that the research was conducted in the absence of any commercial or financial relationships that could be construed as a potential conflict of interest.

## Publisher’s note

All claims expressed in this article are solely those of the authors and do not necessarily represent those of their affiliated organizations, or those of the publisher, the editors and the reviewers. Any product that may be evaluated in this article, or claim that may be made by its manufacturer, is not guaranteed or endorsed by the publisher.
